# Biomarkers of Sepsis-Induced Acute Kidney Injury

**DOI:** 10.1155/2018/6937947

**Published:** 2018-04-24

**Authors:** Kaifei Wang, Sheling Xie, Kun Xiao, Peng Yan, Wanxue He, Lixin Xie

**Affiliations:** ^1^Department of Pulmonary & Critical Care Medicine, Chinese PLA General Hospital, 28 Fuxing Road, Beijing 100853, China; ^2^Nankai University School of Medicine, 94 Weijin Road, Tianjin 300071, China

## Abstract

Sepsis, an infection-induced systemic disease, leads to pathological, physiological, and biochemical abnormalities in the body. Organ dysfunction is caused by a dysregulated host response to infection during sepsis which is a major contributing factor to acute kidney injury (AKI) and the mortality rate for sepsis doubles due to coincidence of AKI. Sepsis-induced AKI is strongly associated with increased mortality and other adverse outcomes. More timely diagnosis would allow for earlier intervention and could improve patient outcomes. Sepsis-induced AKI is characterized by a distinct pathophysiology compared with other diseases and may also have unique patterns of plasma and urinary biomarkers. This concise review summarizes properties and perspectives of the biomarkers for their individual clinical utilization.

## 1. Introduction

Sepsis is a serious medical condition and is caused by a dysregulated host response to infection. Despite advances in antibiotic therapy and life support, the fatality rate of patients with sepsis has remained at least 25% and is increasing in incidence [1]. Sepsis is the most important cause of AKI in critically ill patients, accounting for 50% or more of cases of AKI in ICUs, which was associated with higher risk of in-hospital mortality [2]. Of 192,980 patients with severe sepsis from 7 US states, 22% had AKI and were associated with a mortality of 38.2% [3]. Early diagnosis of sepsis-induced AKI will allow for appropriate and timely interventions that may contribute to significant decreases in the incidence of AKI [4]. Biomarkers, which were recently introduced in many medical fields, including sepsis or AKI, could contribute to the prompt identification of disease. But if we focus on biomarkers of sepsis-induced AKI that have been used in clinical or experimental studies may have a better evaluate of their utility.

The recognition of sepsis dates back to 2000 years, when Hippocrates claimed that sepsis was the process by which flesh rots and wounds fester [5]. After the rapid development of microbiology in the modern era, the understanding of sepsis gradually matured and medical researchers began to realize that sepsis is caused by microbial infection [6]. In the contemporary age, sepsis is generally considered an infection-induced systemic disease that leads to pathological, physiological, and biochemical abnormalities in the body. To date, three versions of a sepsis definition have been proposed and, in 1991, sepsis was defined for the first time as the systemic inflammatory response to infection at the American College of Chest Physicians (ACCP)/Society of Critical Care Medicine (SCCM) conference. Sepsis associated with organ dysfunction is referred to as severe sepsis. Sepsis with sustained hypotension that is difficult to correct after secondary fluid resuscitation is known as septic shock [7]. In 2001, the definition of sepsis was revised by the SCCM/European Society of Intensive Care Medicine (ESICM)/ACCP/American Thoracic Society (ATS)/Surgical Infection Society (SIS), which added specific indicators for inflammation, hemodynamics, organ dysfunction, and tissue perfusion [8]. In 2016, the diagnostic criteria for sepsis were revised and updated by the Sepsis Definitions Task Force of experts from the SCCM/ESICM based on a retrospective analysis of clinical big data. According to the new criteria, clear or suspicious infection and a Sequential Organ Failure Assessment (SOFA) score ≥ 2 are considered the main criteria for the diagnosis of sepsis [9]. Currently, approximately 19 million new cases of sepsis occur worldwide every year [10]. The management of sepsis has long been the focus of research efforts. In 2001, the ESICM, the American Society of Critical Care Medicine, and the International Sepsis Forum launched the “Surviving Sepsis Campaign” (SSC). In 2003, 44 experts among the SCC members developed the treatment guidelines for sepsis, namely, the SSC guidelines based on evidence-based sepsis research. After its first release in 2004, the SSC guidelines were revised in 2008, 2012, and 2016 [11–14]. Owing to the popularization and implementation of the SSC guidelines, the mortality of severe sepsis has been reduced year by year; however, it still reaches up to 28.4% [15].

AKI is defined as “an abrupt and persistent decline in renal function.” In 2002, the Acute Dialysis Quality Initiative (ADQI) AKI RIFLE classification criteria were defined; the access criteria was as follows: creatinine rose to or more than 1.5 times the original or GFR decreased by >25%, or urine output < 0.5 ml/kg/h and continued for more than 6 hours, depending on severity and AKI duration was divided into three levels: risk, injury, failure, and 2 prognosis: loss and end-stage renal disease [16]. In 2004, the American Society Of Nephrology (ASN), the International Society of Nephrology (ISN), and the National Kidney Foundation (NKF), as well as the ADQI and ESICM, convened the meeting to establish AKIN, aimed at establishing a unified definition and classification of AKI standards. In September 2005, the first AKIN conference held in Amsterdam focused on developing uniform standards for the definition and classification of AKI based on the RIFLE standard. Proposed diagnostic criteria for AKI was an abrupt (within 48 h) reduction in kidney function defined as an absolute increase in serum creatinine level of *⩾*26.4 *μ*mol/l (0.3 mg/dl) or a percentage increase in serum creatinine level of *⩾*50% (1.5-fold from baseline) or a reduction in urine output (documented oliguria of <0.5 ml/kg/h for >6 h) [17] (Table 1).

In 2012, the Kidney Disease: Improving Global Outcomes (KDIGO) group performed a retrospective analysis on clinical trials published before 2011 based on the kidney risk, injury, failure, loss, and end-stage renal disease (RIFLE) and Acute Kidney Injury Network (AKIN) criteria. Combined with the evidence in evidence-based medicine, the KDIGO published the KDIGO guidelines in March 2012 and established the diagnostic criteria for acute kidney injury (AKI) [18]: an increase in serum creatinine of >0.3 mg/dl (26.5 *μ*mol/L) within 48 h; or an increase in serum creatinine to 1.5 times baseline, which is known or presumed to have occurred within 7 d; or urine output < 0.5 ml/(kg·h) for 6–12 h. According to the severity, the condition is divided into stages 1, 2, and 3, similar to the AKIN staging; however, the KDIGO staging clarified the admittance criterion, and the diagnostic criteria for each stage are more explicit (Table 2).

## 2. Methods

Based on the published research before March 2017 in Pubmed, Embase, and Cochrane Central Register of Controlled Trails (CENTRAL) databases, a comprehensive computer search was conducted. The keywords we used were “acute kidney injury”, “sepsis”, “biomarkers”, and the names of the biomarkers. After the results have been collated and analyzed, we classified biomarkers of acute kidney injury according to the mechanism and the injury site of AKI. More importantly, we selected the markers that have an early diagnostic value for the sepsis-induced acute kidney injury and summarized them in the hope of helping in choosing the best management in early clinical stage.

## 3. Discussion

### 3.1. Biomarkers of Acute Kidney Injury

The KDIGO guidelines highlight early AKI diagnosis and treatment, and the diagnostic marker remains at serum creatinine level. Because the serum creatinine test is convenient and inexpensive, it provides a practical clinical indicator. However, some limitations exist. Renal hypoperfusion due to a prerenal cause may lead to an increase of creatinine, despite noninjured renal parenchyma [19]. When the renal parenchyma is injured, renal compensation may lead to a lag in the creatinine increase; moreover, injury of 50% of the kidney may occur without an increase in creatinine levels [20, 21], resulting in delayed diagnosis and intervention [22]. Thus, new markers with higher sensitivity and specificity are expected to aid the early diagnosis of AKI. Currently, numerous studies reporting early diagnostic markers of AKI exist, some of which are clinical trials showing good sensitivity and specificity, with early diagnostic value for AKI. Moreover, different biomarkers have clearly been shown to indicate varying mechanisms of injury [22, 23]. The biomarkers of AKI shown in Tables 3 and 4 have been clinically proven to provide certain value for clinical applications.

Evidently, AKI occurs via complex mechanisms often due to multiple factors. Different mechanisms lead to injury in different parts of the kidney. Using the same marker to diagnose injury in all renal subregions caused by all diseases is problematic for establishing a clear diagnosis and accurate injury localization. Discrete study on a specific disease and its associated kidney injury will certainly increase the diagnostic accuracy. Approximately 45–70% of AKI is associated with sepsis, which is one of the most important causes of AKI [2, 24]. Furthermore, the proportion of septic patients with secondary renal injury is 16–50%, while the mortality of sepsis associated with AKI is up to 50–60% [25, 26]. Thus, the focused study of sepsis-induced AKI and search for biomarkers associated with early diagnosis will aid in solving the important clinical problems of sepsis and AKI disease.

### 3.2. The Mechanism of Sepsis-Induced AKI and Biomarkers

During the course of sepsis, immune disorders occur in the body, namely, the imbalance between proinflammatory and anti-inflammatory regulation mechanisms [27]. To maintain homeostasis, the innate immune system can rapidly identify and respond to danger signals, including pathogen-associated molecular patterns (PAMPs) and danger-associated molecular patterns (DAMPs) [28]. After detection by pattern recognition receptors (PRRs), the danger signals activate signaling pathways and induce inflammation through protein receptors (most commonly toll-like receptors), thereby producing early proinflammatory cytokines (TNF-*α* and IL-1*β*) and activating other white blood cells. Neutrophils play a major role in tissue injury. After activation, neutrophils interact with the microvascular endothelium and migrate out of blood vessels to produce superoxide. The superoxide may react with the nitric oxide (NO) produced under induction by mitochondrial NO synthase to form peroxynitrite salt [29], which directly impairs the mitochondrial electron transfer function. Moreover, the superoxide may activate poly (ADP-ribose) polymerase (PARP) to inhibit adenosine triphosphate (ATP) production in mitochondria. This sequence leads to insufficient cellular oxygen utilization in the setting of an adequate tissue oxygen supply, which may eventually cause cell dysfunction and death, thereby impairing organ function [30]. PAMPs are derived from pathogens, while DAMPs are widely produced in injured and infected tissues. These two factors reach the kidney through the circulatory system. DAMPs may also be produced directly from ischemic renal tissue [31]. DAMPs may cause activation of renal vascular endothelial cells and renal tubular epithelial cells, leading to upregulation of adhesion molecules, release of more proinflammatory mediators and reactive oxygen species (ROS), platelet activation and aggregation, microvascular dysfunction, hypoxia, and tissue injury [32]. PAMPs and DAMPs that reach the glomerular filtrate through the circulatory system may affect renal tubular function. Renal tubular epithelial cells prevent renal ischemia-reperfusion injury by upregulating the synthesis and releasing protective proteins, such as neutrophil gelatinase-associated lipocalin (NGAL). Some of the renal tubular cells entering the proliferative cell cycle arrest under stress, thereby reducing energy consumption [33]. Cell cycle arrest may be the primary stage of AKI development in a mouse model of sepsis [34]. In sepsis, cytokine-mediated NO synthesis may reduce systemic vascular resistance and lead to arterial vasodilatation, which may be an important cause of insufficient renal perfusion in sepsis [35]. Maintaining hemodynamic stability may prevent the development of sepsis-induced AKI [36].

### 3.3. Recent Advances in Biomarkers of Sepsis-Induced Acute Kidney Injury

Some of the factors generated in the above processes represent biomarkers for predicting sepsis-associated AKI. Here, we summarize the studies of specific biomarkers for AKI secondary to sepsis:

*sTREM-1*. sTREM-1 is the soluble form of the triggering receptor expressed on myeloid cells 1 (TREM-1). TREM-1, first reported by Bouchon et al. in 2000, is selectively expressed on the surface of neutrophils and monocytes. TREM-1 is a receptor of the immunoglobulin superfamily associated with the inflammatory response, with a relative molecular weight of 26 kDa [37]. TREM-1 may magnify the inflammatory response activated by microbes and their products. Extracellular bacteria and fungi, lipoteichoic acid (LTA), and lipopolysaccharide (LPS) may increase the expression of TREM-1 in phagocytic cells. However, TREM-1 expression is almost undetectable in noninfectious inflammation, such as psoriasis, ulcerative colitis, and vasculitis, despite the increased neutrophil levels [38]. sTREM-1 may be formed after cleavage of the extracellular domain of TREM-1 by metalloproteinases [39]. When AKI occurs, sTREM-1 may be detected in the patient's urine; however, the sTREM-1 level is not related to its serum content, suggesting a potential function of sTREM-1 release in renal injury [40]. Given the above characteristics, sTREM-1 is a candidate biomarker of sepsis-induced AKI, which was verified in clinical trials. In 2011, Su et al. described for the first time the role of urine sTREM-1 in predicting sepsis-associated renal injury [41]. In 2015, a clinical trial by Dai et al. suggested that both serum and urine sTREM-1 levels have diagnostic value for sepsis-induced AKI [42]. 

*NGAL*. Neutrophil gelatinase-associated lipocalin (NGAL) is a glycoprotein composed of 178 amino acid residues. NGAL is a member of the lipid carrier protein superfamily with a molecular weight of 25 kDa and is expressed on the surface of neutrophils [43]. NGAL is also expressed at a low constant rate in a variety of cell types, such as nephrocytes, hepatocytes, and epithelial cells. Therefore, low NGAL levels may be detected in the systemic circulation [44]. NGAL is filtered through the glomeruli and reabsorbed in the proximal tubules [45]. NGAL gene expression was rapidly upregulated in a mouse model of renal ischemia-reperfusion injury [46]. The NGAL expression in the kidney with ischemic injury was significantly upregulated within 3 h of injury and the maximum expression level of NGAL mRNA increased more than 1000-fold 24–48 hours after injury [47]. Thus, NGAL may be used to diagnose AKI, particularly infection-mediated AKI [48]. Clinical studies have shown that serum and urine NGAL levels are elevated in septic patients, which has predictive value for the development of AKI [42, 49]. A meta-analysis of 12 clinical trials that recruited 1582 patients with sepsis found that serum NGAL was applicable for diagnosing sepsis-associated AKI in adults, with a sensitivity of 0.881 (95% CI, 0.819–0.923) and a specificity of 0.474 (95% CI, 0.367–0.582) [50]. Another meta-analysis of 12 clinical studies that included 1263 patients with sepsis concluded that the sensitivity and specificity of urine NGAL for diagnosing sepsis-associated AKI were both 0.80 (95% CI, 0.77–0.83) [51]. A prospective RCT study suggested that urine NGAL was more valuable than serum NGAL in patients with sepsis [52]. 

*Cell Cycle Arrest Protein*. Tissue inhibitor metalloproteinase-2 (TIMP-2) is a 21-kDa nonglycosylated protein comprising 194 amino acid residues, and it regulates cell growth and apoptosis [53]. Urine insulin-like growth factor-binding protein 7 (IGFBP7) is a 29-kDa glycoprotein, a member of the urine insulin-like growth factor-binding protein (IGFBP) superfamily, and is also known as IGFBP-related protein [54]. IGFBP7 expression may be observed in the small intestine, colon, ovary, prostate, testis, spleen, heart, kidney, and pancreas [55]. In patients with AKI, TIMP-2 and IGFBP7 expression are increased in renal tubular cells, which may lead to G1 cell cycle arrest through the induction of p27KIP1 and p21, respectively [56, 57]. This is a response mechanism for early AKI; therefore, the development of AKI may be predicted by testing for TIMP-2 or IGFBP7. In a mouse model of sepsis, TIMP-2 and IGFBP7 were shown to predict the development of AKI, with an area under the receiver operating characteristic curve (AUC) of 0.76 and 0.72, respectively; the result for the [TIMP-2]·[IGFBP7] complex was 0.89 (95% CI, 0.80–0.98) [58]. A multicenter prospective study of 232 patients with sepsis at 39 ICUs in Europe and North America found that the urine test of [TIMP-2]·[IGFBP7] might accurately predict the development of AKI in septic patients, with an AUC of 0.84 (95% CI, 0.77–0.90) in the summary receiver operating characteristic analysis [59]. In 2014, the association of TIMP-2 and IGFBP7 with the development of AKI was supported by the results of two clinical trials of more than 500 critically ill patients at 23 hospitals in the United States. The FDA in the United States examined and approved the safety and efficacy of TIMP-2 and IGFBP7 as risk assessment tools for AKI, and the tests were given marketing approval. This was the first time that the United States allowed the laboratory-to-clinical application use of biomarkers for risk prediction of AKI [60]. 

*KIM-1*. Kidney injury molecule-1 (KIM-1) is a type 1 transmembrane glycoprotein. KIM-1 expression is upregulated in renal proximal tubular cells in ischemic and nephrotoxic AKI [61]. The extracellular fragment of KIM-1 may be shed from proximal tubule cells and detected by immunological methods [62]. A meta-analysis of 11 clinical trials with a total of 2979 patients suggested that AKI prediction using urine KIM-1 had a sensitivity of 74.0% (95% CI, 61.0–84.0%) and a specificity of 86.0% (95% CI, 74.0–93.0%); the summary receiver operating characteristic analysis showed an AUC of 0.86 (95% CI, 0.83–0.89) [63]. However, a stratified analysis of the septic cases was not performed in the previous study. A clinical prospective study of 150 patients with sepsis found that the early diagnosis of sepsis-induced AKI using the urine KIM-1 level at 24 h after admission had an AUC of 0.912 [64]. 

*Netrin-1*. According to its initial description, netrin-1 may affect axonal migration and central nervous system development in the course of neurogenesis [65]. The kidney is the organ with the highest expression level of netrin-1, and the early detection of netrin-1 in urine has been observed in renal tubular ischemia-reperfusion injury [66]. A study of cardiopulmonary bypass (CPB)-associated AKI found that urinary netrin-1 increased 2 h after CPB, peaked at 6 h (2462 ± 370 pg/mg creatinine) and continued to increase up to 48 h after CPB, whereas the increase of creatinine was detected only after 48 h [67]. A study reported that the AUC of urinary netrin-1 reached 0.858 (95% CI, 0.826–0.891) in patients with sepsis-associated AKI [64].

## 4. Conclusion

All of the above biomarkers have been validated using large clinical sample sizes, which have efficient and reliable predictive value for sepsis-associated AKI. Impaired kidney function is well known to be undetectable until more than 50% of the renal parenchyma has been injured. The aforementioned biomarkers appeared in renal parenchyma injury, which is more conducive to early diagnosis, on account of creatinine, and its similar markers are not abnormal until renal function is impaired. Should these biomarkers enter clinical use, they would permit early detection of structural injury within the renal parenchyma, with no need to await the emergence of kidney dysfunction. This prospect is favorable for early intervention, such as the withdrawal or reduced use of nephrotoxic drugs. However, how might clinicians proceed after finding increased levels of these biomarkers? Might the increased level of a particular biomarker, which indicates the opening of a particular signaling pathway by sepsis, guide clinical targeted therapy? Might the increase of these indicators guide the implementation of hemopurification interventions? These are questions to which research must be directed in the next steps toward biomarker clinical utility.

## Figures and Tables

**Table 1:** AKIN criteria definition and classification of AKI.

Stage	Serum creatinine and urine output criteria
1	Serum creatinine increase of *⩾*26.4 umol/L (0.3 mg/dl) or to 150–200% of baseline or urine output < 0.5 ml/kg/h for >6 h
2	Serum creatinine increases to >200–300% of baseline or urine output < 0.5 ml/kg/h for >12 h
3	Serum creatinine increases to >300% of baseline or serum creatinine *⩾*354 umol/L (4 mg/dl) with an acute rise of at least 44 umol/L (0.5 mg/dl) or urine output < 0.3 ml/kg/h for 24 h or anuria for 12 h

**Table 2:** KDIGO criteria definition and classification of AKI.

Stage	Serum creatinine and urine output criteria
1	Serum creatinine increased 1.5–1.9 times baseline or increase >26.4 umol/L (0.3 mg/dl) or urinary output < 0.5 ml/kg/h during a 6 hour block
2	Serum creatinine increased 2.0–2.9 times baseline or urinary output < 0.5 ml/kg/h during two 6 hour blocks
3	Serum creatinine increased >3 times baseline or increased to >353 umol/L (4 mg/dl) or initiation of renal replacement therapy or urinary output < 0.3 ml/kg/h during more than 24 hours or anuria for more than 12 hours

**Table 3:** Different renal injury mechanisms and biomarkers.

Kidney injury mechanism	Biomarkers
Ischemia	Kim-1, NGAL, MCP-1, and cyr61
Hypoxia	L-FABP
cell-cycle arrest	IGFBP 7, TIMP-2

**Table 4:** Different renal injury site and biomarkers.

Kidney injury site	Biomarkers
Glomerular	Urine: TP (total protein), *β*2-microglobulin, Albumin, and *α*1-microglobulin
	Blood: creatinine, cystatin C, and NGAL
Proximal tubules	Kim-1, NAG, netrin-1, IL-18, L-FABP, NET-3, HGF, IGFBP 7, and TIMP-2
Distal tubules	NGAL, GST-*α*/*π*, cystatin C, Cyr61, and NET-3
Collecting duct	Calbindin D28

## Abbreviations

AKI: Acute kidney injury
ACCP: American College of Chest Physicians
SCCM: Society of Critical Care Medicine
ESICM: European Society of Intensive Care Medicine
ATS: American Thoracic Society
SIS: Surgical Infection Society
SOFA: Sequential Organ Failure Assessment
SSC: Surviving Sepsis Campaign
ADQI: Acute Dialysis Quality Initiative
RIFLE: Risk of renal dysfunction, injury to the kidney, failure of kidney function, loss of kidney function, and end-stage kidney disease
ASN: American Society of Nephrology
ISN: International Society of Nephrology
NKF: National Kidney Foundation
KDIGO: The Kidney Disease: Improving Global Outcomes
AKIN: Acute Kidney Injury Network
PAMPs: Pathogen-associated molecular patterns
DAMPs: Danger-associated molecular patterns
NO: Nitric oxide
PARP: Poly (ADP-ribose) polymerase
ATP: Adenosine triphosphate
ROS: Reactive oxygen species
LTA: Lipoteichoic acid
LPS: Lipopolysaccharide
CPB: Cardiopulmonary bypass
sTREM-1: Soluble triggering receptor expressed on myeloid cells 1
NGAL: Neutrophil gelatinase-associated lipocalin
TIMP-2: Tissue inhibitor metalloproteinase-2
IGFBP7: Urine insulin-like growth factor-binding protein 7
KIM-1: Kidney injury molecule-1
MCP-1: Monocyte chemoattractant protein-1
cyr61: Cysteine-rich protein 61
L-FABP: Liver fatty acid binding protein
NAG: N-Acetyl-*β*-glucosaminidase
NET-3: Na+/H+ exchanger isoform 3
HGF: Hepatocyte growth factor
GST-*α*/*π*: *α* and *π* glutathione S-transferases.
